# Robotic-Assisted Stereoelectroencephalography: A Systematic Review and Meta-Analysis of Safety, Outcomes, and Precision in Refractory Epilepsy Patients

**DOI:** 10.7759/cureus.47675

**Published:** 2023-10-25

**Authors:** Fernando De Nigris Vasconcellos, Timoteo Almeida, Augusto Müller Fiedler, Hayes Fountain, Guilherme Santos Piedade, Bernardo A Monaco, Jonathan Jagid, Joacir G Cordeiro

**Affiliations:** 1 Vivian L. Smith Department of Neurosurgery, UTHealth (University of Texas Health) Houston, Houston, USA; 2 Department of Neurosurgery, University of Miami, Miami, USA; 3 Department of Radiation Oncology, University of Miami, Miami, USA; 4 Department of Neurological Surgery, University of Miami Hospital, Miami, USA; 5 Department of Neurological Surgery, University of Miami, Miami, USA; 6 Department of Neurological Surgery, CDF (Clinica de Dor e Funcional), Sao Paulo, BRA; 7 Department of Neurological Surgery, University of Sao Paulo, Sao Paulo, BRA

**Keywords:** drug-resistant epilepsy, operative time, accuracy, robot-assisted, stereoelectroencephalography (seeg)

## Abstract

Robotic assistance in stereoelectroencephalography (SEEG) holds promising potential for enhancing accuracy, efficiency, and safety during electrode placement and surgical procedures. This systematic review and meta-analysis, following Preferred Reporting Items for Systematic Reviews and Meta-Analyses (PRISMA) guidelines and International Prospective Register of Systematic Reviews (PROSPERO) registration, delves into the latest advancements and implications of robotic systems in SEEG, while meticulously evaluating outcomes and safety measures. Among 855 patients suffering from medication-refractory epilepsy who underwent SEEG in 29 studies, averaging 24.6 years in age, the most prevalent robots employed were robotic surgical assistant (ROSA) (450 patients), Neuromate (207), Sinovation (140), and ISys1 (58). A total of 8,184 electrodes were successfully implanted, with an average operative time of 157.2 minutes per procedure and 15.1 minutes per electrode, resulting in an overall mean operative time of 157.7 minutes across all studies. Notably, the mean target point error (TPE) stood at 2.13 mm, the mean entry point error (EPE) at 1.48 mm, and postoperative complications occurred in 7.69% of robotically assisted (RA) SEEG cases (60), with 85% of these complications being asymptomatic. This comprehensive analysis underscores the safety and efficacy of RA-SEEG in patients with medication-refractory epilepsy, characterized by low complication rates, reduced operative time, and precise electrode placement, supporting its widespread adoption in clinical practice, with no discernible differences noted among the various robotic systems.

## Introduction and background

Epilepsy is a prevalent neurologic condition, affecting an estimated 50 million people worldwide, and it is characterized by recurrent, unprovoked seizures. While most patients can manage their seizures pharmacologically, some individuals develop medication-refractory epilepsy, which is defined as a persistent seizure disorder despite the adequate use of two combined anticonvulsant agents [1].

The epileptogenic zone (EZ) is defined as the first site where seizures begin and are organized [2]. For patients with refractory epilepsy, surgical resection of the EZ is the treatment of choice to achieve seizure control [3-5]. In many cases, the EZ may contain functional regions of the brain that control eloquent or important functions. Thus, it is critical to precisely determine the EZ location and boundaries during procedures to avoid postoperative neurological complications [5-7].

This necessity for extremely precise localization drove Jean Talairach and Jean Bancaud to create the procedure known as stereoelectroencephalography (SEEG) between 1950 and 1960 in France. As an invasive approach for investigating medication-resistant focal epilepsies, SEEG enables the creation of a precise three-dimensional analysis of epileptiform discharges in a temporal manner. The procedure involves the placement of multi-lead depth electrodes into the cortical and subcortical regions of interest. The combination of SEEG monitoring data with clinical observations allows for more precise identification of seizure onset zones, subsequently enabling more precise targeting of resection zones.

Since its conception, it has taken decades for SEEG to be implemented worldwide. The ability to target deep brain structures while keeping the rate of complications low has made SEEG the current gold standard for surgical planning and resection of EZs. Current indications for SEEG use include investigation of deep cortical and sulcal structures, recording from both hemispheres and three-dimensional (3D) mapping of EZs. Given the valuable information provided by this technique, non-invasive methods are now used in a complementary manner [8-11].

However, with technological advancements in neurosurgery such as improved imaging modalities, stereotactic guidance, and robot-assisted navigation, SEEG has been steadily accruing recognition. Similarly to robotic systems across all fields of medicine and neurosurgery in particular, the use of robots in SEEG procedures has gained significant national and international attention. Researchers are investigating robotic-assisted techniques to improve accuracy, minimize invasiveness, and enhance the overall efficiency of the approach to the target resection zone [9,12].

This systematic review and meta-analysis aims to thoroughly examine recent literature concerning the utilization of various robotic systems in SEEG procedures for patients with medication-refractory epilepsy. The primary focus of this study is the comparative analysis of these systems, evaluating their accuracy, complication rates, and operative time. The goal is to expand the current body of knowledge concerning the safety and effectiveness of RA-SEEG, while also delving into its potential benefits and constraints.

## Review

Methods

Review Record and Search for Studies

The systematic review was conducted in accordance with the Preferred Reporting Items for Systematic Reviews and Meta-Analyses (PRISMA) protocol and registered on the International Prospective Register of Systematic Reviews (PROSPERO) platform under the registration ID CRD42023434560. Two independent authors performed sensitive searches on the MEDLINE database to select the articles used. Studies published until May 2023, when the last survey was carried out for article collection, were considered for inclusion. The search strategy used is displayed in Table 1.

**Table 1:** Search Strategy for Databases

Search Strategy	Database
(Robotic SEEG [Title/Abstract]). (Robot assisted stereoelectroencephalography [Title/Abstract]). ((Robotic [Title/Abstract]); OR (Robot assisted [Title/Abstract]); AND ((SEEG [Title/Abstract]); OR (Stereoelectroencephalography [Title/Abstract]).	PubMed, PubMed Central (PMC), and Medline

Inclusion Criteria

Two independent authors utilized the Covidence software to screen the search results obtained from two databases following pre-established inclusion and exclusion criteria. The studies selected for inclusion satisfied the following criteria: 1. reported using robotic systems for SEEG; 2. reported outcomes of the procedure and its complications; and 3. prospective or retrospective studies. We excluded studies that met the following criteria: 1. case reports; 2. absence of abstract or full paper; and 3. non-English studies.

Data Extraction

The following data was extracted from the included studies: study design, the number of patients who were treated, age, sex, robotic system used, operative time, total number, and mean number of placed SEEG electrodes, time per electrode, entry point error (EPE), target point error (TPE), invasive monitoring duration, intraoperative complications, and postoperative complications.

Risk of Bias Assessment

The evaluation of potential bias in the included studies was performed using a widely accepted tool for appraising the quality of case series studies [13]. Two independent reviewers evaluated each study's risk of bias, and any discrepancies were resolved through thorough discussion.

Statistical analysis

To provide descriptive statistics, counts (N, %) and means (range) were employed while adopting medians for variables that displayed a non-normal distribution. Single-arm meta-analysis was performed using the software IBM SPSS Statistics Data Editor version 29th. Standard deviations were calculated according to the Wan et al. model [14] and later transformed into standard errors. Comparisons using the following variables were done: EPE, TPE, and total operative time. p-values were calculated with a 95% confidence interval (CI), and p<0.05 was used to determine statistical significance. Q-test of subgroup homogeneity was calculated. Studies that did not provide any dispersion measures were excluded from the meta-analysis.

Results

Descriptive Analysis

**Figure 1:**
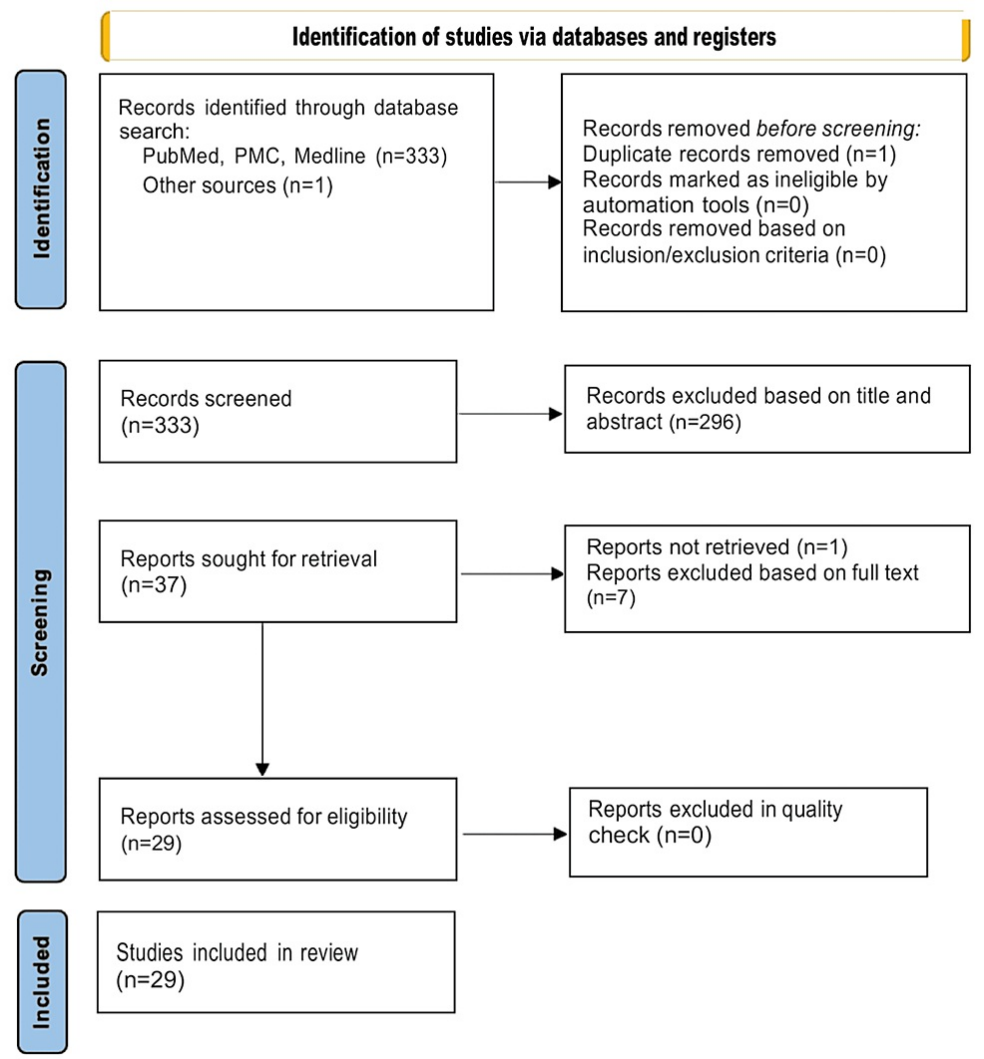
PRISMA Flowchart PRISMA: Preferred Reporting Items for Systematic Reviews and Meta-Analyses, PMC: PubMed Central.

This review included data from 855 patients from 29 studies who underwent robotically assisted (RA) SEEG for refractory epilepsy (Figure 1) [15-44]. Table 2 illustrates the assessment of bias risk for included studies.

**Table 2:** Risk of Bias Assessment

	Selection	Ascertainment		Causality				Reporting	Total
		Exposure	Outcome	Alternative causes ruled out	Challenge-rechallenge phenomenon	Dose-response effect	Adequate follow-up		
Nelson et al. 2020 [41]	+	+	-	+	+	+	+	+	7+
De Benedictis et al. 2017 [38]	+	+	+	+	+	+	+	+	8+
Miller et al. 2017 [40]	+	+	+	+	+	+	+	+	8+
Chaitanya et al. 2020 [37]	+	+	-	+	+	+	-	+	6+
Ollivier et al. 2017 [35]	+	+	+	+	+	+	+	+	8+
Bourdillon et al. 2018 [36]	+	+	+	+	+	+	-	-	6+
Bonda et al. 2021 [42]	+	+	+	+	+	+	+	+	8+
Abhinav et al. 2013 [43]	-	+	-	+	+	+	-	-	4+
Vakharia et al. 2021 [25]	+	+	+	+	+	+	+	-	7+
Urgun et al. 2021 [19]	+	+	+	+	+	+	-	+	7+
Lu et al. 2021 [16]	+	+	-	+	+	+	-	-	5+
Dorfer et al. 2017 [29]	+	+	+	+	+	+	-	+	7+
Kandregula et al. 2021 [17]	-	+	-	+	+	+	-	-	4+
Spyrantis et al. 2019 [22]	-	+	-	+	+	+	-	-	4+
Candela-Cantó et al. 2018 [32]	+	+	+	+	+	+	+	+	8+
González-Martínez et al. 2016 [26]	+	+	+	+	+	+	+	+	8+
Spyrantis et al. 2018 [27]	+	+	-	+	+	+	+	-	6+
Minchev et al. 2022 [33]	-	+	-	+	+	+	-	+	5+
Kalbhenn et al. 2021 [44]	+	+	+	+	+	+	+	+	8+
Iordanou et al. 2019 [21]	-	+	-	+	+	+	-	-	4+
Liu et al. 2023 [39]	+	+	-	+	+	+	-	+	6+
Yao et al. 2023 [15]	+	+	+	+	+	+	-	+	7+
Zheng et al. 2021 [18]	+	+	+	+	+	+	+	+	8+
Ho et al. 2018 [23]	+	+	+	+	+	+	+	+	8+
Kim et al. 2020 [28]	+	+	+	+	+	+	-	+	7+
Abel et al. 2018 [34]	+	+	-	+	+	+	-	-	5+
Zhao et al. 2020 [31]	+	+	-	+	+	+	+	+	7+
Machetanz et al. 2021 [30]	+	+	+	+	+	+	+	+	8+
Bottan et al. 2020 [24]	+	+	-	+	+	+	-	+	6+

From all studies included, 450 patients were treated with the robotic surgical assistant (ROSA) system; 207 with Neuromate; 140 with Sinovation; and 58 with ISys1. The age of the population, including both pediatric and adult populations, was available in 25 papers. The mean age was 24.6 years, ranging from two to 64 years old. Gender was described for 678 patients, 358 males (52.5%) and 320 females (47.5%).

The number of SEEG electrodes placed was available for 811 patients, and the total number was 8,184, with a mean of 10.09 per patient, ranging from 1 to 21. Specifically, the ROSA system was responsible for 4,848 (59.2%) electrodes in 411 patients; Neuromate for 1,585 (19.3%) electrodes in 202 patients; Sinovation for 1,330 (16.3%) electrodes in 140 patients; and ISys1 for 421 (5.2%) in 58 patients.

The average duration of electrophysiologic monitoring after the procedures was 8.3 days in 289 patients from 11 studies. Regarding operative times, 589 patients had an available total operative time (mean: 157.2 minutes, range: 71 to 414 minutes), while 584 had a mean time per electrode (mean: 15.1 minutes, range: 6.3 to 37 minutes). With respect to the different robots, ROSA's mean operative time was 147.7 minutes and 11.45 minutes per electrode. Neuromate’s mean total operative time was 257 minutes and 36.6 minutes per electrode. Sinovation’s mean operative time was 114.8 minutes and 9.9 minutes per electrode. ISys1's mean total time was 135.3 minutes and 15.7 minutes per electrode. Table 3 summarizes the mean operative times and the total number of SEEG electrodes.

**Table 3:** Operative Times and Number of Electrodes ROSA: robotic surgical assistant.

	Overall	ROSA	Neuromate	Sinovation	ISys1
Total number of electrodes	8,184 (811 patients)	4,848 (411)	1,585 (202)	1,330 (140)	421 (58)
Mean number of electrodes	10.06 (811)	11.8 (411)	7.8 (202)	9.5 (140)	7.25 (58)
Total operative time (minutes)	157.2 (589)	147.7 (338)	257 (93)	114.8 (126)	135.3 (32)
Time per electrode (minutes)	15.1 (584)	11.45 (338)	36.6 (88)	9.9 (126)	15.7 (32)

The accuracy of these procedures was defined through entry point error (EPE) available for 616 patients and target point error (TPE) available for 666 patients, both measured in millimeters. The mean overall EPE was 1.48 mm (range: 0 to 8.37 mm). Regarding different robotic systems, ROSA accounted for 286 patients, with a mean EPE of 1.49 mm (range: 0.3 up to 6.38 mm). Neuromate had data available for 152 patients, with an overall mean EPE of 1.6 mm (range: 0 to 8.37 mm). Sinovation described a mean EPE of 1.39 mm, with no reported range, for 120 patients. ISys1 had 58 patients with a mean EPE of 1.23 mm (range: 0.1 to 3.4 mm).

In terms of target point error (TPE), the average measurement across all patients was 2.13 mm (range: 0 to 7.3 mm). For the ROSA robot, data was available for 336 patients, and the average TPE was 2.50 mm (range: 0 to 9.02 mm). Neuromate was utilized in 152 patients, with an average TPE of 2.09 mm (range: 0 to 7.3 mm). Sinovation was employed in 120 patients, resulting in an average TPE of 1.64 mm (range: 0.33 to 3.61 mm). ISys1 was used in 58 patients, and the average TPE was 1.61 mm (range: 0.3 to 6.7 mm) (Table 4).

**Table 4:** EPE and TPE NA: not available, EPE: entry point error, TPE: target point error.

	Overall	ROSA	Neuromate	Sinovation	ISys1
Mean EPE (mm)	1.48 (616 patients)	1.49 (286)	1.66 (152)	1.39 (120)	1.23 (58)
Range EPE (mm)	0-8.37 (616)	0.3-6.38 (268)	0-8.37 (152)	NA	0.1-3.4 (58)
Mean TPE (mm)	2.13 (666)	2.5 (336)	2.09 (152)	1.64 (120)	1.61 (58)
Range TPE (mm)	0-7.3 (666)	0-9.02 (336)	0-7.33 (152)	0.33-3.61 (120)	0.3-6.7 (58)

Data was available for 708 patients regarding intraoperative complications, and not a single case was reported in which the procedure itself had to be altered in any way due to unforeseen complications. Hemorrhages diagnosed in the immediate postoperative period were classified as postoperative complications in these studies. Data on this type of complication was reported by 24 papers, which included 780 patients. The total number of cases was 60, with an overall risk of postoperative complications of 7.69%. Only nine (15%) cases were symptomatic and required further treatment. The complications reported were 51 cases of hemorrhage (85% of total complications, with 46 intracerebral and five extra-axial); three cases of edema (5% of total complications); three episodes of infection (5% of total complications) with two being infections of the surgical site and one meningitis; two (3.3%) cases of pneumocephalus; and one (1.7%) case of Stroke-like Migraine Attacks after Radiation Therapy (SMART) syndrome, upper extremity deficits. No permanent deficits were noted, and all symptomatic complications had full recovery to baseline.

With respect to complications for the different robotic systems: ROSA had data for 380 patients of which 36 had complications (9.47% risk of complication), Neuromate had complications in 10 patients out of 202 total patients (4.95% risk of complication), Sinovation had 12 cases in 140 total patients (8.57% risk of complications), and ISys1 had two complications in 58 total patients (3% risk of complications).

Meta-Analysis

**Figure 2:**
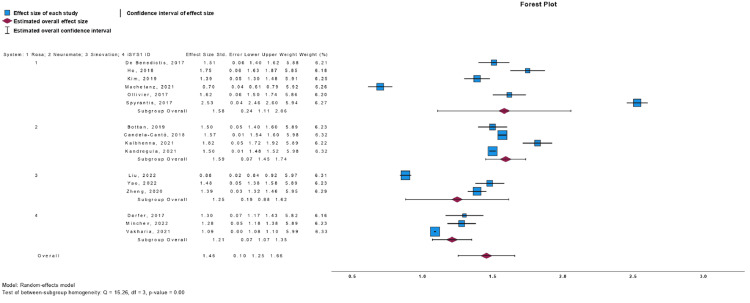
EPE Forest Plot EPE: entry point error. Sources from De Benedictis et al. [38], Ho et al. [23], Kim et al. [28], Machetanz et al. [30], Ollivier et al. [35], Spyrantis et al. [27], Bottan et al. [24], Candela-Cantó et al. [32], Kalbhenna et al. [21], Kandregula et al. [17], Liu et al. [39], Yao et al. [15], Zheng et al. [18], Dorfer et al. [29], Minchev et al. [33], and Vakharia et al. [25].

**Figure 3:**
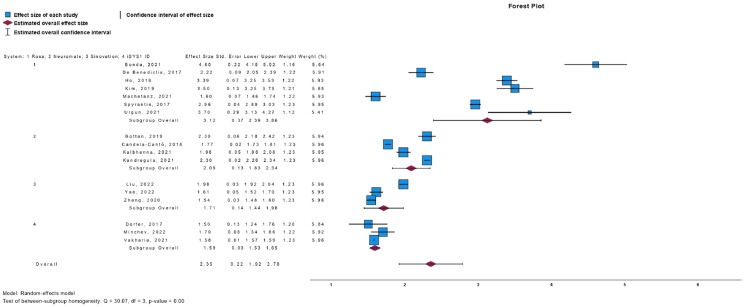
TPE Forest Plot TPE: target point error. Sources from Bonda et al. [42], De Benedictis et al. [38], Ho et al. [23], Kim et al. [28], Machetanz et al. [30], Spyrantis et al. [27], Urgun et al. [19], Bottan et al. [24], Candela-Cantó et al. [32], Kalbhenna et al. [21], Kandregula et al. [17], Liu et al. [39], Yao et al. [15], Zheng et al. [18], Dorfer et al. [29], Minchev et al. [33], and Vakharia et al. [25].

**Figure 4:**
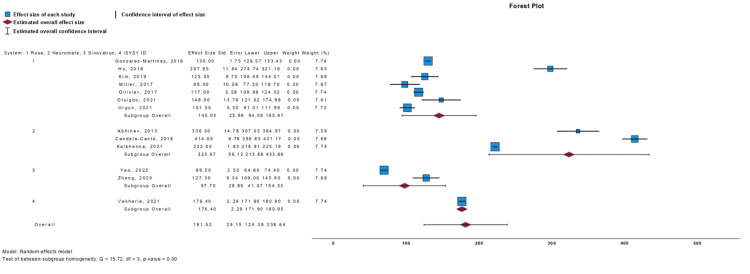
Operative Time Forest Plot Sources from Gonzalez-Martínez et al. [26], Ho et al. [23], Kim et al. [28], Miller et al. [40], Ollivier et al. [35], Oluigbo et al. [41], Urgun et al. [19],  Abhinav et al. [43], Candela-Cantó et al. [32], Kalbhenna et al. [21], Yao et al.[15], Zheng et al. [18], and Vakharia et al. [25].

We conducted single-armed meta-analyses to compare the endpoints of EPE, TPE, and operative time across various robotic systems. The results are presented in Figures 2-4 for EPE, TPE, and operative time, respectively. In terms of EPE, all the different robotic consoles exhibited overlapping intervals of accuracy, indicating no statistically significant differences among them. Conversely, for TPE values, the ROSA robot demonstrated higher values and wider intervals compared to the other systems, but no difference with the overall analysis. Regarding operative time, the neuromata system exhibited the highest values, which did not overlap with those of other systems individually. However, the overall analysis did not reveal a significant difference.

Discussion

Epilepsy is a neurological disorder that can significantly impact the quality of life. Patients with epilepsy often experience recurrent seizures, leading to physical injuries, cognitive impairment, and social isolation. While there are several medical treatment options available for epilepsy, a subset of these epilepsy patients will develop medication-refractory epilepsy and may not have clear benefits from medications. Surgical resection of epileptogenic loci is then the treatment of choice for medication-refractory epilepsy. Given the novelty of using robots in SEEG, there has been a surge of recent studies investigating the potential benefits and limitations of this technique. Our review gathers and compiles the relevant data from these studies in order to provide a comprehensive overview of the current literature. Here, we will organize our discussion and analysis around three key aspects of robot-assisted SEEG: operative time, accuracy, and safety. We will also address the limitations of this study. To the best of our knowledge, this is the first study that objectively compares how different robotic systems perform.

Operative Time

Overall, the average total operative time was 157.2 minutes and the time per electrode was 15.1 minutes. These findings align closely with recent studies that demonstrate a reduction in the duration of the operation associated with robotically assisted (RA) procedures in comparison to traditional approaches [45,46]. In a recent systematic review by Gomes et al., the time per electrode reported was 7.36 minutes and 23.32 minutes for RA and manually guided interventions, respectively [47]. Our findings fall within the range observed in these studies, supporting the current literature on the improvement of time while using robots.

In our data, the Neuromate system showed the longest surgical time and time per electrode with 257 and 36.6 minutes, respectively. All other systems had total operative times performed in less than 150 minutes. A possible reason for this discrepancy may be the team’s learning curve. As illustrated in some studies, the mean operative time in the initial cases was considerably longer than the final cases, suggesting a possible learning curve effect.

Prolonged operative times have been shown to be strongly associated with an increased risk of both surgical site and central nervous system (CNS) infections. Given these potential consequences, optimizing surgical time without increasing risks is of paramount importance. Additionally, minimizing the length of post-durotomy operative time reduces cerebrospinal fluid (CSF) leak, further decreasing the likelihood of anatomical distortion and brain shift [48-52].

Accuracy

As previously extensively explained by Vakharia et al., EPE refers to the difference between the actual and planned position of the electrode when passing through the skull and can be influenced by misregistration of the neuronavigation system, inaccurate alignment, and deflection during drilling [48]. Distinct from EPE, TPE is the difference between the actual and planned position of the electrode at the target site and can be affected by various factors such as the angle of entry, electrode deflection, electrode rigidity, and depth of insertion. It is believed that the insertion technique has a more significant impact on EPE. At the same time, the system's stability affects the angle of entry, which in turn impacts TPE accuracy. It is believed that when compared to the traditional manually guided techniques, robots can increase accuracy rates to less than 2 mm [53].

In our study, all papers that reported their EPE and TPE had means within the current literature. Bonda et al. reported having 80% of their trajectories within a 1 mm distance of the plan [42]. When analyzing the subgroup meta-analyses for these values, both showed ROSA having higher means and intervals of accuracy, which is likely due to the fact that it is the most widely used console worldwide, and obviously, different groups have different backgrounds and expertise. For both measures, iSYS1 was the robot with the lowest error measures, but it was used in a smaller number of procedures, which might be the reason for the lowest error measures. In general, no statistically significant differences were observed among the various systems.

Furthermore, some previous studies assess how robots perform versus manually guided SEEG. A meta-analysis by Philipp et al. suggests that the use of robots independently contributes to the decrease in TPE [45]. A famous large cohort study by Cardinale et al. reported a mean EPE of 1.43 mm and TPE of 2.69 mm for operations using the traditional manually guided SEEG approach among 419 procedures [46]. A systematic review by Vakharia et al. compared both methods and showed no statistical significance between them [48]. More recently, Gomes et al. compared four different studies and reported a significant mean reduction in EPE of 0.57 mm when using RA compared to manually guided SEEG [47]. With respect to TPE, they found no statistical difference between the techniques.

Complications

A systematic review by Mullin et al. had the evaluation of SEEG’s safety as their primary objective [10]. They thoroughly studied complications by following 22,085 electrodes that were implanted in 2,624 patients. According to their data, SEEG has an overall complication rate of approximately 1.3% and it has one of the lowest complication rates of all invasive monitoring procedures [54].

The most common complication reported in different SEEG series is intracranial hemorrhages. Fortunately, most of the reported intracranial hemorrhages are small and asymptomatic. In the study by Mullin et al., only 11 out of the 2,624 patients had to have surgical evacuation of the hematoma due to its size [10]. In their study, 0.6% of the patients had permanent neurological deficits following SEEG [10].

In our analysis of the data, we observed the incidence of hemorrhages as the most common complication, representing about 85% of the observed complications (51 cases). Of these cases, the majority were asymptomatic. In our analysis, no patients had permanent neurologic deficits following the procedure. No meta-analyses were performed assessing complications across the different robotic systems, due to the lack of data for these calculations.

The recent systematic review by Gomes et al. recorded nine out of 145 (6.2%) intracranial hemorrhages in the RA method versus eight out of 139 (5.7%) in the manually guided group, with no statistically significant difference [46]. No difference was observed for other complications such as infections and permanent neurological deficits.

Limitations

The retrospective nature and case-series design of some of the papers included in our study constitute important limitations. Additionally, the use of different robotic systems by different surgical teams with varying levels of expertise presents a potential confounding factor when analyzing variables of interest such as operative times and accuracy. The lack of standardization in the reporting of accuracy data across studies, including the use of different metrics and methods (such as Euclidean distance, 2D error, radial error, 3D error, mean, median, and interquartile range), further limits the ability to draw meaningful conclusions from the available data.

## Conclusions

SEEG is a safe and effective procedure that significantly improves the precision of resection zones in patients with medication-refractory epilepsy. Importantly, it achieves this without increasing the risks of complications during or after surgery, all while consistently maintaining high levels of accuracy. Our research indicates that when it comes to factors such as accuracy and the time taken for the procedure, there are no significant differences among the various robotic consoles used in SEEG. However, it is important to highlight that future studies that consider the costs associated with each of these devices could offer a more comprehensive understanding of their cost-effectiveness. This additional information could be instrumental for healthcare professionals when making decisions about which robotic system to utilize in SEEG procedures.
